# Discrepancies Between Self-reported and Objectively Measured Smartphone Screen Time: Before and During Lockdown

**DOI:** 10.1007/s10935-023-00724-4

**Published:** 2023-01-24

**Authors:** Pedro B. Júdice, Eduarda Sousa-Sá, António L. Palmeira

**Affiliations:** 1 CIDEFES, Universidade Lusófona, Lisbon, Portugal; 2 Early Start, University of Wollongong, Wollongong, NSW Australia; 3 Faculty of Sport, Research Centre in Physical Activity, Health and Leisure, University of Porto, Porto, Portugal

**Keywords:** Assessment, Screen time, Physical activity, Sedentary behavior, Validity

## Abstract

Screen time shows higher health risks compared to other types of sedentary behaviors. A lockdown may simultaneously increase screen time, reduce physical activity (PA), and change time perception. Our goal was to compare self-reported against objectively measured smartphone screen time (SST) in a sample of active and inactive Portuguese adults before and during a social lockdown. This study was a cross-sectional analysis with 211 Portuguese adults (57.8% males), aged 25.2 ± 8.5 years, from two cohorts, one before the social lockdown and the other during the lockdown. SST was self-reported (SR-SST) and objectively measured using a smartphone (OM-SST). PA was self-reported. Linear regressions were performed to determine the association between SR-SST and OM-SST. A Bland and Altman analysis was used to assess agreement. Independent T-tests were performed for comparisons between cohorts and paired sample T-tests for comparisons within each cohort. The cohort assessed during the lockdown showed a higher SST than the cohort assessed before the lockdown (OM-SST; *p* < 0.001 and SR-SST; *p* = 0.009). Before the lockdown, there was no difference between SR-SST and OM-SST (*p* = 0.100). However, during the social lockdown, although the agreement between SR-SST and OM-SST was good (ICC = 0.72), participants systematically underestimated their SST by ~ 71 min/day (*p* < 0.001), and this underestimation was higher in inactive participants (~ 85 min/day) than in active individuals (~ 49 min/day). The general population needs to be aware of the benefits of limiting screen time, especially during periods of societal modifications, such as a generalized lockdown. There was a tendency to underestimate SST, meaning a lack of awareness of the actual time spent in this potentially deleterious behavior. This underestimation was more pronounced during the lockdown period and for the inactive participants, thus posing a greater health risk. The findings from this investigation entail relevant information for policy makers to delineate strategies for reducing population screen time from a preventive health perspective.

## Introduction

Sedentary behavior (SB) refers to any waking behavior characterized by an energy expenditure of less than 1.5 METs in a seated/reclined position (Tremblay et al., 2017), with excessive time in this behavior being associated with several diseases (Patterson et al., 2018; Wilmot et al., 2012) and even mortality (Chau et al., 2013; Patterson et al., 2018). SB encompasses different types of activities, such as the more traditional ones (e.g., watching television and working on the computer), and other modern behaviors, such as smartphone screen time (SST). Although specific SB entails distinct associations with health parameters, overall screen time is often the one associated with higher health risks (Arango et al., 2014; Biddle et al., 2017; Hu et al., 2003; Wijndaele et al., 2011).

Research has shown, quite consistently, that increased screen time (from 3 to 4 h/day) is associated with multiple adverse health outcomes, including obesity, type 2 diabetes (Dunstan et al., 2010), depression (Wang et al., 2019), and increased risk of all-cause mortality, irrespective of physical activity (PA) levels (Celis-Morales et al., 2018). Also, the number of deaths assigned to this unhealthy lifestyle is belligerently increasing in adults (Pratt et al., 2020; Saint-Maurice et al., 2020). Moreover, reduced PA and increased SB might lead to an increase in body weight and impairment of glycemic control, which in turn can trigger a lipid profile disturbance and increased inflammation and cardiometabolic risk (Martinez-Ferran et al., 2020). Hence, finding efficient and scalable measures of overall screen time could provide crucial data to prevent several health problems, both at the individual level (e.g., as a self-monitoring tool) and at the governmental level (e.g., by asking individuals to easily share their data with policymakers via automated processes, the so called “passive data”).

With technological development, smartphones have become instruments for communication that allow people to do a wide range of tasks such as work and shopping, amongst others. This potentially makes smartphones the primary source of daily screen time, which has been increasingly integrated into everyday life (Vizcaino et al., 2020). Findings on objectively measured SST build upon prior work based on self-report and confirm that adults spend a considerable amount of time using their smartphones (Christensen et al., 2016). Furthermore, smartphone usage differs across ages, but is similar across socio-economic groups. In adults, it seems to be associated with lower PA and high SB (Grimaldi-Puyana et al., 2020; Mack et al., 2021). Finally, smartphone overuse negatively affects mental health (Jeong et al., 2020; Mack et al., 2021), and can even change some cortical processes in the brain (Gindrat et al., 2015).

Although evidence suggests that self-reported SB is lower when compared to sensor-based data (Chastin et al., 2018; Prince et al., 2018), it still remains a reasonably good method to assess SB in large scale studies (Ishii et al., 2018; Sudholz et al., 2018; Wijndaele et al., 2014). To the authors' knowledge, no investigation has analyzed the ability of people to self-report the amount of time spent in SST against the actual time. Modern smartphone operating systems now present data on the users' daily screen time. This information can be harvested and correlated with self-reported measures. If correlated, it will provide a future pragmatic solution for gathering data about SB, which could be used in prevention interventions at a large scale.

Due to the COVID-19 pandemic, Portugal, as in many other countries, implemented physical distancing measures, such as school and business closures, and the restriction of people to leave their houses outside of essential medical care, food, or medicine shopping in order to slow down the spread of COVID-19. It is well known that this pandemic encompassed some behavioral changes [i.e., higher SB, lower PA, and social lockdown (DGS, 2020)], which could potentially be associated with higher smartphone usage (Mack et al., 2021). In fact, recent evidence suggests that people increased their usage of digital media during the lockdown (Mack et al., 2021). Sleep timing during the lockdown also seemed to change, with people going to bed and waking up later, and reporting a lower sleep quality (Cellini et al., 2020). But more interestingly, this increase in sleep difficulties was associated with the feeling of elongation of time (Cellini et al., 2020). Comparisons between self-reported and objectively measured sleep time suggest that lockdown restrictions can affect sleep onset and wake-up time perception, which indicates that the ability of a person to accurately perceive the time spent in any behavior, including SST, may be jeopardized during a lockdown (He et al., 2021). Home confinement reduces the exposure to daylight and increases the level of stress due to social isolation and the impossibility to engage in satisfying activities, which can also impact the pace of the flow of time (Zakay, 2014).

Furthermore, there is evidence suggesting that during lockdown, not only time perception (He et al., 2021), but also time allocation may have changed according to the habitual PA levels (Sarangi et al., 2022). One investigation showed that college students using active modes of transportation before lockdown allocated less time to SB than physical activities during the lockdown period, and their perception of leisure time activities was significantly different from those using private and public modes of transportation (Sarangi et al., 2022). Another study with university students found that, during the COVID-19 lockdown, people spent more time on their smartphones, were more sedentary, visited fewer locations, and exhibited increased symptoms of anxiety and depression (Mack et al., 2021). Thus, considering that SST is expected to increase during a lockdown and based on the assumption that both PA levels and the lockdown itself may be associated with time perception, our investigation aimed to compare self-reported against objectively measured SST in a sample of Portuguese adults from two cohorts, one before the COVID-19 lockdown and the other during the COVID-19 lockdown, and to analyze if PA levels and the lockdown context were associated with the participants' ability to self-report SST. Our hypotheses are that (1) the usual underestimation of SST will be higher in a lockdown context and (2) this underestimation will be higher in inactive participants than in active individuals.

## Materials and Methods

### Study Design and Sample Recruitment

The study comprised of two cross-sectional groups with 211 Portuguese adults from two cohorts. This study included a convenience sample of university students, teachers, and relatives, recruited through social networking and snowball effect. Participants were recruited if they were aged between 18 and 64 years old, owned a smartphone encompassing an app to objectively measure SST and had no other constraints. Cohort 1 (n = 65) was evaluated between January and February of 2019 (i.e., prior to the first COVID-19 lockdown in Portugal), and the second cohort (n = 146) was assessed between January and February 2021 (i.e., during the second COVID-19 lockdown in Portugal). The same online questionnaire was applied to both cohorts, and participants responded to it autonomously and anonymously. This investigation was submitted to the Ethics Committee of the University, but as this was an observational study, ethical approval was not compulsory. Nevertheless, it was conducted according to the Declaration of Helsinki and a detailed description of the study procedures was provided to each participant, and an informed consent was obtained by each participant in the online questionnaire prior to participation in the study.

### Measures

#### Demographics and the Assessment of Self-reported and Objective Smartphone Screen Time

To ask the participants about their perceived SST, we developed a specific question: "Please think about the last 7 days, how much time did you spent using your smartphone on average per day?". This question was in a separate section of the online questionnaire where participants could not return and change their initial response in order to avoid participants going to their smartphones and seeing the objectively measured SST. After this question, participants were asked about their age, sex, weight, height, occupation, and operating system of their smartphones (i.e., iOS or Android). Participants' body mass index (BMI) was calculated as weight/height^2^ (kg/m^2^) and were then categorized according to the World Health Organization (WHO) cut points, as normal weight (BMI < 25) and overweight/obese (BMI ≥ 25). At the end of the questionnaire, there was a simple script explaining the steps that the participant would have to follow to have access to the objectively measured daily average SST based on the last 7 days (i.e., specific scripts for the iOS and Android operating systems), and the participants only had to write down the value presented on the screen of their smartphone.

#### Physical Activity Assessment

Two simple questions were asked to estimate moderate and vigorous physical activity (MVPA) considering the prior 7 days. The first question asked how many days per week the participant performed any kind of MVPA (i.e., frequency) and the second question asked the average length of MVPA per day (i.e., duration per day). The frequency was multiplied by the daily duration to obtain the total weekly volume of MVPA performed in the previous 7 days. As an anchor so that the participants better understood what MVPA meant, the same definitions used in the International PA Questionnaire (IPAQ) were used; “Moderate activities refer to activities that take moderate physical effort and make you breathe somewhat harder than normal, and vigorous physical activities refer to activities that take hard physical effort and make you breathe much harder than normal”. Based on this variable, participants were categorized as physically active (≥ 150 min/week) or inactive (< 150 min/week), based on the current WHO PA guidelines (Bull et al., 2020).

### Statistical Analysis

Descriptive statistics (means ± *SD*) were calculated for all continuous variables. Normality was tested using Shapiro–Wilk test, and this assumption was verified. Group comparisons between self-reported and objectively measured SST were analyzed using paired sample T-test. Independent T-tests assessed differences between the two cohorts for the main variables, and the Levene's test showed equality of variances. Cohen’s *d* effect size was calculated, and the following criteria were used; small (*d* = 0.2), medium (*d* = 0.5), and large (*d* = 0.8) (Cohen, 1988). Linear regressions were performed to calculate the association between self-reported SST and objectively measured SST. As concordance correlation coefficient is defined without ANOVA assumptions, we calculated the intraclass correlation coefficient (ICC), which has been traditionally used for assessing reliability between multiple methods. We calculated ICC using an absolute agreement definition for the two-way mixed models available on SPSS.

Agreement between methods was assessed using the Bland and Altman approach (Bland & Altman, 1986), including the 95% limits of agreement. Bland and Altman quantifies the difference between measurements using a graphical method. A scatterplot is drawn in which the X-axis represents the average [(K1 + K2)/2], and the Y-axis represents the difference (K1–K2) of two measurements. The mean bias (mean of the K1–K2) and its confidence limits (limits of agreement) should be quantified. An ideal agreement is zero difference between measurements. In a good agreement, the scattering of points is diminished, and points lie relatively close to the line which represents the mean bias (Doğan, 2018). The association between the mean and the difference of both methods is tested to indicate the trend. If there is an association, it means that the difference between methods is not constant, and if no association exists, a constant error is found, meaning that a correction factor can be possibly applied.

In addition to the Bland and Altman analysis, we performed linear regression models between the self-reported and objectively measured SST, which allowed us to visually assess the precision (i.e., Pearson correlation coefficient). For accuracy, we used a bias correction factor that measured how far the best-fit line deviates from the identity line through the origin. Data analyses were performed using IBM SPSS Statistics version 25.0 (SPSS Inc., an IBM Company, Chicago, Illinois, USA) for the entire sample and based on (1) before COVID-19 lockdown *vs.* during COVID-19 lockdown; (2) complying *vs.* not complying with the MVPA recommendations. Since no interaction was found for sex with the differences between methods, the analyses were not performed separately for sex. For all tests, statistical significance was set at 5%.

## Results

This study included 211 participants (57.8% males), from which 30.8% were analyzed before the COVID-19 lockdown, and 69.2% were analyzed during the lockdown. Considering the operative system of their smartphones, 27.5% were Android, and 72.5% were iOS. More than half of the sample (53%) did not comply with the MVPA recommendations. Finally, 71.1% had normal weight.

Table 1 presents the overall sample's characteristics and by cohort.

**Table 1:** Participants‘ characteristics for the overall sample and stratified by cohort

	Overall mean (*SD*)	Pre-lockdown mean (*SD*)	During-lockdown mean (*SD*)	*p*-value*
	(*n* = 211)	(*n* = 65)	(*n* = 146)	
Age (years)	25.2 (8.5)	24.4 (8.5)	25.5 (8.5)	0.386
Weight (kg)	67.1 (13.7)	66.5 (13.7)	67.3 (13.7)	0.691
Height (cm)	168.6 (9.0)	169.6 (9.5)	168.1 (8.8)	0.286
BMI (kg/m^2^)	23.5 (3.7)	23.0 (3.5)	23.7 (3.8)	0.191
SR-SST (min/day)	211.7 (124.6)	178.2 (105.8)	226.6 (129.7)	**0.009**
OM-SST (min/day)	266.7 (173.7)	198.2 (107.5)	297.2 (188.5)	**< 0.001**
MVPA (min/week)	182.5 (202.8)	234.5 (198.4)	159.3 (201.1)	**0.013**
*p*-value^#^	**< 0.001**	0.100	**< 0.001**	

The bold means a significant difference

*n* number of participants, *SD* standard deviation, *BMI* body mass index, *MVPA* moderate-to-vigorous physical activity, *OM-SST* objectively measured smartphone screen time, *SR-SST* self-reported smartphone screen time

*p-value for the independent sample T-test

^#^*p*-value for the paired sample T-test regarding the comparison between self-reported and objectively measured smartphone screen time

As shown in Table 1, the two cohorts did not differ in age, weight, height, and BMI (*p* > 0.05). However, the independent T-tests showed that the pre-COVID-19 cohort presented significantly lower self-reported (*p* = 0.009) and objectively (*p* < 0.001) measured SST and higher MVPA (*p* = 0.013) than the COVID-19 cohort.

Moreover, at the group level, there was no difference between self-reported and objectively measured SST in the cohort assessed prior to the COVID-19 lockdown (mean difference = 20.0 min/day; *p* = 0.100). However, in the cohort assessed during the COVID-19 lockdown, a group difference was found between self-reported and objectively measured SST (mean difference = 70.6 min/day; *p* < 0.001), with the self-reported method underestimating the objectively measured SST.

Table 2 presents the main characteristics according to the MVPA categories for the overall sample and stratified by cohort.

**Table 2:** Smartphone screen time and MVPA for the overall sample and by MVPA categories, stratified by cohort

	Overall mean (*SD*)	Pre-lockdown mean (*SD*)	During-lockdown mean (*SD*)	*p*-value*	Cohen’s d
Inactive	(*n* = 111)	(*n* = 24)	(*n* = 87)		
SR-SST (min/day)	215.2 (130.3)	194.4 (126.0)	220.9 (131.6)	0.380	− 0.203
OM-SST (min/day)	283.4 (173.0)	201.5 (113.8)	305.9 (180.1)	**0.008**	− 0.620
MVPA (min/week)	36.7 (46.5)	51.9 (49.2)	32.5 (45.1)	0.070	0.422
*p*-value^#^	** < 0.001** (− 0.495)	0.742 (− 0.068)	** < 0.001** (− 0.601)		

Active	(*n* = 100)	(*n* = 41)	(*n* = 59)		
SR-SST (min/day)	207.8 (118.5)	168.7 (92.4)	235.0 (127.4)	**0.003**	− 0.580
OM-SST (min/day)	248.2 (173.3)	196.2 (105.0)	284.3 (201.1)	**0.005**	− 0.522
MVPA (min/week)	344.4 (185.9)	341.5 (172.9)	346.4 (195.9)	0.898	− 0.026
*p*-value^#^	**0.001** (− 0.329)	0.063 (− 0.299)	**0.009** (− 0.352)		

The bold means a significant difference

*n* number of participants, *SD* standard deviation, *BMI* body mass index, *MVPA* moderate-to-vigorous physical activity, *OM-SST* objectively measured smartphone screen time, *SR-SST* self-reported smartphone screen time

*p-value for the independent sample T-test

^#^*p*-value for the paired sample T-test regarding the comparison between self-reported and objectively measured smartphone screen time

As shown in Table 2, for the inactive participants, the two cohorts only differed in the objectively measured SST (*p* = 0.008), with the pre-COVID-19 cohort presenting lower objectively measured SST. However, for the active group, there were differences between the two cohorts for both the self-reported (*p* = 0.003) and objectively measured SST (*p* = 0.005), with the pre-COVID-19 cohort presenting lower values.

At the group level, there was no difference between self-reported and objectively measured SST in the cohort assessed prior to the COVID-19 lockdown for both the inactive (mean difference = 7.1 min; *p* = 0.742) and active (mean difference = 27.5 min; *p* = 0.063) participants. However, in the cohort assessed during the COVID-19 lockdown, differences were found between self-reported and objectively measured SST in both inactive (mean difference = 85.1 min; *p* < 0.001) and active participants (mean difference = 49.2 min; *p* = 0.009), with the self-reported method underestimating SST.

**Fig. 1:**
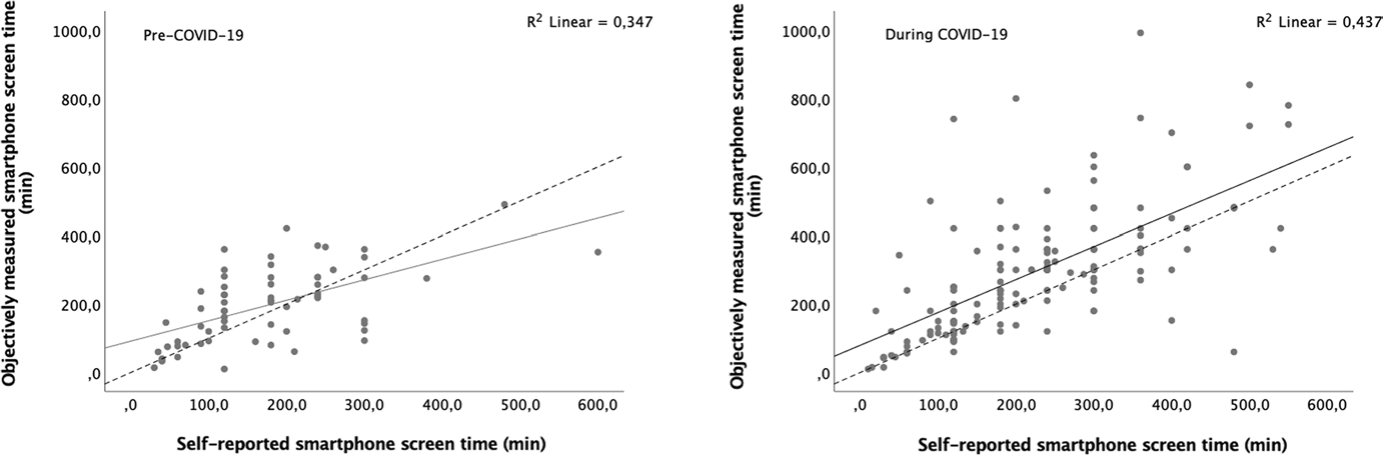
Regression analysis for the self-reported with objectively measured smartphone screen time, in both cohorts. *The dashed line represents the identity line and the continuous line the regression line

Figure 1 depicts the association between self-reported and objectively measured SST in both cohorts. For the pre-COVID-19 cohort, there was an association between self-reported and objectively measured SST (β = 0.598; *p* < 0.001). The self-reported method explained 37% of the objectively measured SST variability after the adjustment for age, sex, BMI, and weekly MVPA. The precision (i.e., Pearson correlation coefficient), which measures how far each observation deviates from the best-fit line, was 0.59 (*p* < 0.001). In terms of accuracy, it is possible to observe that both the slope and the intercept of the best-fit line were different from the identity line (95% confidence interval: 48.9–134.3 for the intercept and 0.39–0.81 for the slope).

For the cohort assessed during the COVID-19 lockdown, there was also an association between self-reported and objectively measured SST (β = 0.961; *p* < 0.001). The self-reported explained 46% of the objectively measured SST variability after the adjustment. The precision was 0.66 (*p* < 0.001), and in terms of accuracy, the intercept of the best-fit line was different from the identity line (95% confidence interval 32.6–126.4), but no difference was found for the slope (95% confidence interval 0.78–1.14).

**Fig. 2:**
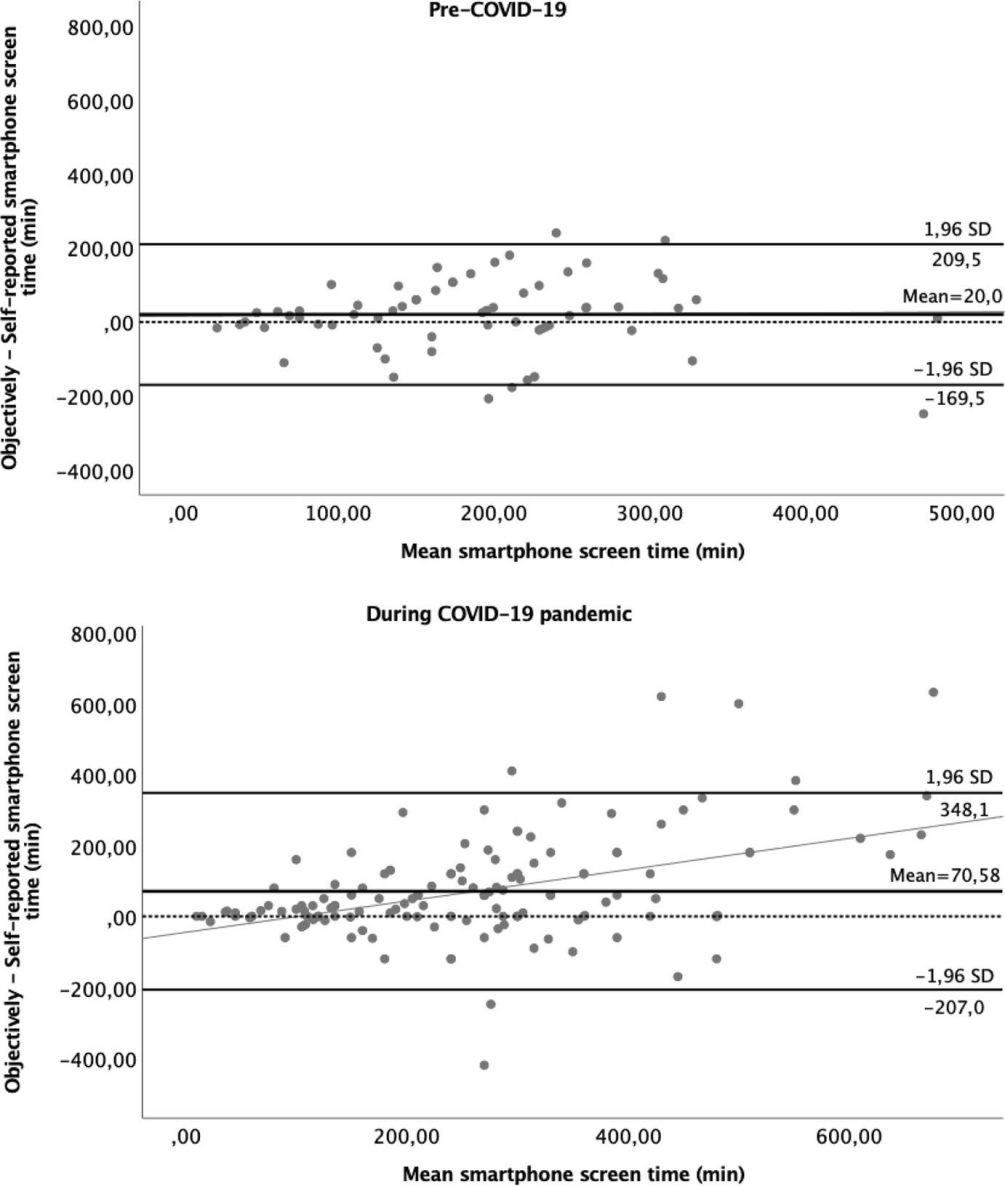
Bland and Altman analysis for the differences between self-reported and objectively measured smartphone screen time, in both cohorts. *The dashed line represents the identity line and the continuous line the regression line

Figure 2 presents the Bland and Altman analyses for both cohorts. These analyses indicated a fixed bias, with the self-reported method systematically underestimating the objectively measured SST by 20 min/day, with no proportional bias but high limits of agreement in the pre-COVID-19 cohort. The trend between the mean and the differences was not significant (slope = 0.019; *p* = 0.881), thus no proportional bias, meaning that the individual's capacity to reliably self-report SST was independent of the SST magnitude. The agreement was good (ICC = 0.74).

In the cohort assessed during the COVID-19 lockdown, the Bland and Altman indicated a proportional bias, with a significant trend between the mean and the difference of the two methods (slope = 0.442; *p* < 0.001), meaning that the greater the SST, the less the individual's capacity to reliably report it. The self-reported method tended to underestimate to a larger extent when more time was spent in SST. The agreement was good (ICC = 0.72).

Finally, the difference between methods was not associated with age, BMI, or weekly MVPA (*p* > 0.05), considering both the overall sample and stratified by cohort.

## Discussion

This work presents a timely investigation of comparisons between self-reported and objectively measured SST in Portuguese adults before and during the COVID-19 lockdown. We observed that participants consistently underestimated SST, especially during the COVID-19 lockdown (~ 71 min/day). No such analysis has been made for SST; therefore, we will compare our results to those obtained for SB. A recent review, which compared self-reported and device measures of SB in adults, found that self-reported measures underestimated SB/sitting time by 1.74 h/day in normal life conditions (Prince et al., 2020). Our results showed a similar trend but with a lower difference, which is natural considering that we examined a specific type of SB, and not the overall SB.

Our findings highlight that during the lockdown, the underestimation was even higher than in normal life conditions, which supports the evidence suggesting that reductions in exposure to daylight from home confinement may not only increase the level of stress due to social isolation (e.g., avoid social contact with family and friends), but can also impair the pace of the time’s flow (He et al., 2021; Zakay, 2014), meaning that the ability to self-report time spent in a specific behavior can be harder (He et al., 2021). Also, the context of a lockdown can generate a feeling of elongation of time (Cellini et al., 2020), which may explain the higher underestimation of SST in the lockdown cohort, when compared to the cohort evaluated before the lockdown. Finally, irrespective of being in a lockdown or not, the higher the time spent in one behavior, the harder it can be to accurately self-report that same behavior (Kim & Welk, 2017). This was confirmed by our data from the lockdown cohort, where the Bland and Altman analysis suggested a higher underestimation from the participants spending longer time in SST (i.e., proportional bias with a significant trend between the mean and the difference of the two methods), meaning that the greater the SST, the less the individual's capacity to reliably report it.

Furthermore, we also believe that the lack of social zeitgebers, such as regular work schedules and social activities, as well as changes in living conditions (e.g., moving to parents' house) during the lockdown may somehow contribute to this impaired sense of time (Erren et al., 2004), which again may jeopardize the ability to accurately self-report SST. Additionally, PA may also play an important role as a social zeitgeber in humans, acting as a cue in the regulation of the body's circadian rhythm (Mistlberger & Skene, 2004). Indeed, during lockdown, when dividing the sample by MVPA categories, our findings showed that those in the inactive category underestimated SST to a larger extent (~ 85 min/day) when compared to the active ones (~ 49 min/day), suggesting that higher PA levels may help the participants to track time better and improve their ability to self-report this specific behavior.

However, although the underestimation of SST was lower in the active participants as compared to the inactive ones, there was still a significant underestimation of this behavior during the lockdown in both these participants. This finding somehow confirms the results from a recent investigation aiming to assess the relationship between subjectively and objectively measured PA levels during COVID-19-based restrictions and after they were lifted, which showed that the ability of active adolescents to perceive their behaviors is impaired during lockdown conditions (Cocca et al., 2021). Furthermore, in our study, the inactive group had ~ 1 h 44 min more SST per day during the COVID-19 lockdown (i.e., objectively measured) than the cohort assessed before COVID-19, whereas differences were smaller for the active group (i.e., ~ 1 h 28 min/day for objectively measured SST).

Besides the fact that participants were experiencing home confinement, the lockdown coincided with the spread of technologies, such as the news feed and attention-grabbing advertising on major social networks, which could explain the higher SST in both active and inactive groups. Again, no investigation has performed these types of analyses, but we believe that for the inactive group, their engagement in more SST compared to the active group is somewhat expected, as screen time is the most prevalent type of SB reported in adults (Castro et al., 2020). Nevertheless, those belonging to the active category, also had their SST increased during the lockdown, which might be related to the steep increase in the use of online home fitness programs through new technologies (videos and apps) as solutions for maintaining activity (Ravalli & Musumeci, 2020).

Evidence suggests that people increased the usage of digital media during lockdown, but also reported changes in their sleep timing and lower sleep quality (Sarangi et al., 2022). This may suggest that, not only time perception, but also time allocation during lockdown may have changed according to habitual PA levels. A study with college students showed that those using active commute modes before lockdown allocated less time to SB and more time to physical activities during the lockdown period, and that students' perception of leisure activities among those who used active commute modes was significantly different from those using private and public modes of transportation (Sarangi et al., 2022). Thus, PA levels seem to be of relevance when studying the ability to self-report SST, and one must expect an underestimation, especially in the inactive participants, and this underestimation should be considered when designing interventions to prevent inactivity-related health issues.

Another potential explanation for the observed differences between self-reported and objectively measured SB has been said to be the recall period in the self-reported method, as studies have found that reducing the recall period may increase the validity and accuracy of self-reported measures for SB (Clark et al., 2013; Matthews et al., 2013). Thus, considering the overall underestimation found in our findings, future investigations should explore shorter self-reporting periods, such as the previous 2 days or the previous day, which may improve precision and accuracy even further, although the agreement found in our data was good (i.e., ICC ranging from 0.72 to 0.74).

As expected, the cohort assessed during the COVID-19 lockdown exhibited higher SST, when compared to the pre-COVID-19 cohort, for both self-reported and objective methods (~ 48 min for self-reported and ~ 1 h 39 min for objectively measured). The higher SST during the COVID-19 lockdown was accompanied by a ~ 1 h 15 min lower MVPA/week. These results align with studies from around the world during the COVID-19 lockdown, showing decreases in PA (Giustino et al., 2020; Qin et al., 2020) and increases in overall screen time (Sun et al., 2020). These results may be explained by a dramatic shift in the normality of the daily routines, as many Portuguese suddenly found themselves in a new working environment (working from home), helping their children with home-schooling, as well as interacting with friends and family online. This change imposed an increase in dependence on smartphones and home internet connections, which was globally reported through an increase in total internet traffic of 40–60% during the spring 2020 global lockdown period (OECD, 2020), whereas before COVID-19 people could rely more on other types of screens and other non-digital sources of information.

Concerning PA, recent evidence showed that prolonged restrictions could lead to decreased opportunities for outdoor exercise (Chen et al., 2020), however, we did not assess the preferred location of our sample for engaging in PA. Nevertheless, and although the WHO made guidelines available to adopt during home confinement (WHO, 2020), as well as the easy access to several online home-based training programs available for free, we can only assume that home confinement and significant circulation restrictions reduced overall PA levels and access to exercise.

Research has shown, quite consistently, that increased screen time (from 3 to 4 h/day) is associated with multiple adverse health outcomes (Dunstan et al., 2010; Wang et al., 2019) and all cause mortality (Celis-Morales et al., 2018), irrespective of PA levels. Altogether, these findings clearly support the need to facilitate and encourage PA, as well as to limit screen time throughout the COVID-19 lockdown or other public health-related restrictions, however long they may be required. Finally, the potentially deleterious effects of home confinement (increased screen time and decreased active time) will probably only be fully seen in the long term. There is a robust health rationale for maintaining PA while at home in order to stay healthy and maintain a proper immune system function in the current hazardous environment. The message “stay at home“ cannot be mistaken for "stay on the couch". In most lockdowns, PA was one of the few allowed outside activities so, a positive, full-of-opportunities message should be used: "doing some PA is better than doing none, everything counts" (Segar et al., 2020).

This study's strengths include data on SST and MVPA pre- and during-COVID-19 public health restrictions measured with two different types of tools (self-reported and objectively measured for the SST). Nonetheless, this study is not without limitations; first the issue with objectively measured SST is that it is still somehow self-reported. We can generally assume that participants were honest and indicated the SST displayed on their smartphones, but as this action was performed autonomously and independently by the participants, there are always issues around social desirability that can somehow impact their answers. However, the differences between self-reported and objectively measured SST indicate that, overall, this may not be the case. Secondly, the cross-sectional design precludes inference of causality, and the sample is predominantly well-educated and Caucasian and not representative of the entire Portuguese population. It is also possible that the association is bi-directional, i.e., people are less willing to engage in PA during the lockdown period, which may have led to an increase on SST. Finally, even though the majority of SST may be spent in SB, one must not disregard that some portion of the SST can be followed by active commuting or ambulating, which does not impact the main purpose of the present investigation but should be highlighted.

The current findings strongly support the implementation of urgent actions to tackle this symbiotic relationship between increased screen time and decreased PA levels. The general population needs to be informed about the benefits of limiting screen time and increasing PA levels, especially during societal modifications due to a world pandemic. This message becomes even more critical due to a steep increase in SST and the tendency to be underestimated by people when self-reporting it. Future research should try to replicate these findings in larger samples and from different cultural backgrounds, investigate potential cross-national differences and include different age ranges, and, ideally, explore longitudinal dynamic associations of SST and PA.

The current movement guidelines clearly state that "Every Move Counts" to protect and achieve a healthy status (Bull et al., 2020). But looking at recent literature, it is also clear that "every no-move counts." Hence, accessing accurate movement measures (including SST) is of utmost importance for individuals, practitioners, and policymakers who strive to improve human health worldwide. Given the wealth of movement and SST data available on our smartphones, we foresee the development of interventions that would inform the individual, practitioners, and policymakers using the same data sources. For example, a shared pool of data, with fewer accuracy-related errors, would allow for interventions that are precise (what content), individualized (who is the recipient of the content), and just-in-time (when), improving their effectiveness both at the prevention and treatment levels.

## Data Availability

The datasets are not publicly available but are available from the corresponding author upon reasonable request.
